# The Impact of Early Bilingualism on Face Recognition Processes

**DOI:** 10.3389/fpsyg.2016.01080

**Published:** 2016-07-19

**Authors:** Sonia Kandel, Sabine Burfin, David Méary, Elisa Ruiz-Tada, Albert Costa, Olivier Pascalis

**Affiliations:** ^1^GIPSA-Lab (CNRS UMR 5216), Université Grenoble AlpesGrenoble, France; ^2^Institut Universitaire de FranceParis, France; ^3^Laboratoire de Psychologie et NeuroCognition (CNRS UMR 5105) – Université Grenoble AlpesGrenoble, France; ^4^Universitat Pompeu FabraBarcelona, Spain; ^5^Institució Catalana de Recerca i Estudis AvançatsBarcelona, Spain

**Keywords:** face processing, Other-Race Effect, bilingual, monolingual

## Abstract

Early linguistic experience has an impact on the way we decode audiovisual speech in face-to-face communication. The present study examined whether differences in visual speech decoding could be linked to a broader difference in face processing. To identify a phoneme we have to do an analysis of the speaker’s face to focus on the relevant cues for speech decoding (e.g., locating the mouth with respect to the eyes). Face recognition processes were investigated through two classic effects in face recognition studies: the Other-Race Effect (ORE) and the Inversion Effect. Bilingual and monolingual participants did a face recognition task with Caucasian faces (own race), Chinese faces (other race), and cars that were presented in an Upright or Inverted position. The results revealed that monolinguals exhibited the classic ORE. Bilinguals did not. Overall, bilinguals were slower than monolinguals. These results suggest that bilinguals’ face processing abilities differ from monolinguals’. Early exposure to more than one language may lead to a perceptual organization that goes beyond language processing and could extend to face analysis. We hypothesize that these differences could be due to the fact that bilinguals focus on different parts of the face than monolinguals, making them more efficient in other race face processing but slower. However, more studies using eye-tracking techniques are necessary to confirm this explanation.

## Introduction

Human social life is built through interactions, achieved via facial expressions and language communication. Pascalis et al. (2014) proposed that face and language processing are intimately linked in their development, as they are part of the social communication system. For example, face recognition processes follow similar perceptual narrowing patterns than visual speech perception. Perceptual narrowing refers to a progression whereby infants maintain ability to discriminate stimuli to which they are exposed, but lose ability to discriminate stimuli to which they are not exposed (for face processing see Kelly et al., 2007; for speech processing see Pons et al., 2009). Adults are experts for the faces and languages present in their environment. Their face and language systems are interactive, and their neural mechanisms are linked to some extent.

However, narrowing can be prevented in infants for experienced classes of visual stimuli (Pascalis et al., 2005; Scott and Monesson, 2009; Heron-Delaney et al., 2011). Maurer (2015) claimed that in some cases narrowing can be disrupted and that a different adult will emerge. In other words, differences in perceptual mechanisms in adults may be a consequence of differences that occurred during the perceptual narrowing processes. Maurer (2015, p. 595) has been studying adults with synesthesia, who may have “undergone less perceptual narrowing.” Adults with synesthesia were better than controls at discriminating between other species faces and discriminating non-native sounds. A change in the development of the visual system has then affected face and language processing abilities. Would a different experience with language during development affect the narrowing of other cognitive functions?

The present study intends to shed some light on the relationship between linguistic experience and face processing. Early Bilinguals grow up hearing/seeing more than one language. They are therefore sensitive to more than one linguistic auditory and visual code. The person with whom they communicate will determine which language they will code. They switch from one code to another after identifying the speaker. One way to do so is by recognizing his/her face. Monolinguals only have one code, so they do not have to relate a person to a specific language code. Does this have an impact on the way Bilinguals and Monolinguals will processes language and faces when they are adults? Our previous research revealed that Monolingual adults were more accurate and faster than Bilinguals when having to identify phonemes in audiovisual presentations but not in audio-only conditions. In the present study we examined whether these differences in visual speech decoding could be due to differences in the visual processing of the speaker’s face. To do so, we compared face processing abilities in Bilinguals and Monolinguals.

Face and language processing are intimately linked (Pascalis et al., 2014). Auditory information alone is of course sufficient to understand speech, but we systematically and unconsciously rely on the visual information provided by the speaker’s face. Seeing the oro-facial gestures of the speaker accelerates word recognition, which is the core process underlying face-to-face conversations (Fort et al., 2010, 2012). In addition, oro-facial information enhances consonant and vowel intelligibility in noisy environments (Sumby and Pollack, 1954; Benoît et al., 1994). During the first year of life, perceptual narrowing in face development happens at the same ages as for visual speech processing. The temporal concomitance of these processes is not surprising since face-to-face communication involves both phoneme and face discrimination abilities. However, the infants’ linguistic experience seems to affect the timing of the perceptual decline for visual speech. There are several studies indicating that infants growing up in a monolingual environment tend to loose language discriminating abilities earlier than the children living in a bilingual environment (e.g., Weikum et al., 2007).

### Bilingual Infants

Early bilingualism affects the infants’ abilities for language detection. Weikum et al. (2007) found that the ability to distinguish French from English in silent videos declined at 8 months for English monolinguals but not for the infants raised in a *bilingual* English–French environment. Sebastián-Gallés et al. (2012) further investigated this “bilingual delay” by presenting Weikum et al.’s (2007) videos to a group of 8-month-old infants who had never heard English or French in their environment. The infants were Spanish monolinguals and Spanish–Catalan Bilinguals. Their data revealed that the *bilingual* Spanish–Catalan infants distinguished the English from French videos whereas the monolinguals did not. This is evidence that the perceptual decline appears later when having to deal with two languages at the same time early in life, irrespective of the familiarity the infant has with these languages. Another study also indicates that the perceptual narrowing phenomenon also applies to sign language (Palmer et al., 2012). At 4 months hearing infants are able to distinguish one sign from another in American Sign Language (ASL). At 14 months they are no longer capable of doing so. Although the signs are physically different, the children at this age tend to confuse them. Conversely, ASL-learning hearing infants –i.e., that grow up in a bilingual environment– are still able to discriminate the signs at 14 months of age. This implies that perceptual language abilities are re-organized during the first year of life irrespective of language support: gesture-oral.

### Bilingual Adults

The “bilingual delay” during the perceptual narrowing period seems to affect the way we process language as adults. Weikum et al. (2013) found that Spanish adults who learned English late had difficulties in distinguishing French/English silent videos compared to participants who learned English early in life. These results suggest that the linguistic experience the participants had during their childhood had a long term impact on the way they processed visual speech, at least when having to discriminate one language from another.

Another research conducted with Monolingual and Bilingual adults provides further support for a link between early linguistic experience and visual speech processing (Burfin et al., 2014). Monolinguals and early Bilinguals had to discriminate a Bengali dental-retroflex phonemic contrast that does not exist in their native language/s. The Bengali phonemes were presented in audio-only or audiovisual conditions. In the audio-only presentation both groups had serious difficulties in discriminating the Bengali phonemes and confused them most of the time. In the Audiovisual presentation both groups took advantage of the visual information on the speaker’s face movements. With the oro-facial information on the speaker’s gestures all the participants could discriminate the dental phonemes from the retroflex ones. However, Monolinguals had a global performance that was 10% higher than Bilinguals. In addition, the “audiovisual benefit” –i.e., accuracy in the audiovisual condition compared to the accuracy in the audio-only condition– was much higher for Monolinguals (22%) than Bilinguals (13%). The authors also observed that Bilinguals were slower to respond correctly than Monolinguals in the audiovisual presentation. Therefore, the early exposure to more than one language can affect the way we take advantage of the visual information on the speaker’s face movements in adulthood.

### Bilingualism and Face Processing

Any face-to-face conversation involves an analysis of the speaker’s face to locate the relevant cues for speech decoding. Visual processes locate the mouth with respect to the eyes, nose, etc. Unlike Monolinguals, Bilinguals also have to identify the speaker to know which language they should decode in visual speech. Face recognition should be determinant in the identification process. In addition, Bilinguals have to associate which oro-facial gestures correspond to which language. These supplementary processes that Bilinguals have to do systematically since their early childhood may modulate the way they process faces and visual speech. This would constitute important processing differences with respect to Monolinguals.

A few studies examined this issue in an adult population, whereas the developmental literature is more consequent (e.g., Pons et al., 2015). Adult data presented by Zhang et al. (2013) revealed that when Chinese immigrants have to speak English their language fluency is modulated by the kind of face they are presented with. Their English is better if the interlocutor’s photograph is Caucasian than when it is Chinese. This suggests that face features –especially the ones that convey information on the origin of the speaker and thus the language that he/she might speak– can have an impact on language processing. Another line of evidence supporting the idea of a link between visual speech decoding and face processing comes from de Heering et al. (2012) who conducted a face processing experiment with deaf adults. In de Heering et al.’s (2012) experiment, the face processing abilities in deaf adults differed from those of matched hearing adults in several ways. The deaf population was more accurate for face recognition than the hearing adults. The inversion effect of the deaf population was larger. The deaf were globally slower than controls. This pattern of results was interpreted as reflecting face scanning strategy differences. Hearing participants tend to privilege the eye area. The deaf participants focused less on the eyes than the controls and might have an enhancement of the visual representation of the mouth (McCullough and Emmorey, 1997). de Heering et al. (2012) concluded that deaf participants probably needed more time to process the same information than the hearing adults when processing inverted faces, enhancing their inversion effect. Thus, they may rely more on configural processing. There is, however, an alternative to explain their results. This population is of particular interest because it acquired lip-reading and sign language simultaneously in early childhood. Lip-reading and sign language are two linguistic codes that convey information through physically different channels. Since these codes obey to the same kind of linguistic structure as any other human language, we may consider this population as bilinguals. They were *early* bilinguals because they learned both codes soon after birth. Their face processing differences might then also be linked to the use of two languages at early stage in their life.

On another perspective, Haussman et al. (2004) investigated hemispheric specialization differences between German Monolinguals and Turkish–German Bilinguals during linguistic and face-discrimination tasks. Their results indicated that Bilinguals do not have the same left visual field advantage than Monolinguals during face discrimination. Bilinguals’ reaction times were longer than Monolinguals’ when faces were presented in the left visual field. This reveals a difference in cortical organization for face processing between the two populations. On this basis, and if Bilinguals and Monolinguals process faces differently, early exposure to several languages can have an impact on their lip-reading abilities.

The aim of the present study was to investigate whether early bilingualism affects face processing mechanisms. To examine this question we used the Other-Race Effect paradigm (ORE). The ORE refers to the difficulty in recognizing and processing faces of members of a race or ethnic group other than our own (Kelly et al., 2007). Experimentally, this phenomenon leads to more recognition errors when a target face is from an unfamiliar racial group rather than our own racial group (see Meissner and Brigham, 2001, for a review). If Bilinguals and Monolinguals use different mechanisms to process faces, we should observe differences in ORE between the two populations. Based on Maurer (2015), we hypothesized that bilinguals’ face narrowing processes may have not happened in the same way as in monolinguals; bilinguals might exhibit a smaller ORE.

We also used picture-plane inversion as a manipulation because the dominant view is that inversion disrupts the ability to perceive a face holistically/configurally. Specifically, it has been suggested that whereas upright faces are encoded as integrated wholes, inverted faces are rather processed feature-by-feature, in a piecemeal manner (e.g., Yin, 1969; Sergent, 1984; Farah et al., 1998; Rossion, 2008; Rossion, 2009). If bilingual participants focus more than monolingual participants on the bottom part of the face (McCullough and Emmorey, 1997), their inversion effect should be limited or absent because the most diagnostic feature of the face to match inverted faces are the eyes. Alternatively, if they process faces relying on global cues, they should exhibit an equally large or even enhanced face inversion effect compared to monolinguals.

## Materials and Methods

### Participants

A total of 41 early Bilinguals participated in the experiment. Information on the participants’ linguistic experience was collected with an adapted version of the Language Experience and Proficiency Questionnaire (Marian et al., 2007). There were 24 Catalan–Spanish Bilinguals (mean age = 20 years). They have all been exposed to both languages from birth. Mean age of acquisition of Spanish and Catalan was 11 and 14 months, respectively. Ten learnt Spanish and Catalan at home. Seven have always been exposed to Spanish but lived in a Catalan environment and seven have been exposed to Catalan from birth but learnt Spanish in nursery school. They were students at the University Pompeu Fabra (Barcelona, Spain). There were also 17 Bilinguals of different languages (mean age = 19.6 years). They spoke French and another language from birth: English (four participants), German (four participants), Italian (four participants), Spanish (three participants), Malagasy (one participant), and Portuguese (one participant). They had all been exposed to both languages from birth. They spoke French because they lived in Grenoble from birth and the second language was their parents’ native one (both mother and father’s mother tongue). They had equivalent verbal fluency in both languages. They were students at the University of Grenoble Alpes or at the Cité Scolaire Internationale which is an international school in Grenoble where only proficient Bilinguals can attend. These Bilingual participants also participated in the experiment on native and non-native phoneme identification presented by Burfin et al. (2014). The Monolingual group consisted of 41 French native speakers (mean age = 22 years). They all learnt English as a second language in middle school but their proficiency was poor. They had no experience in a foreign country of more than 1 month. They were students at the University of Grenoble Alpes (Grenoble, France) and received course credit for participation. All the participants –Monolinguals and Bilinguals– gave written consent for participating in the experiment. The Bilinguals of the Cité Scolaire Internationale also provided parental consent to participate in the experiment. The method of this study is in agreement with the ethical guidelines of the ethical committee for Cognitive Science experiments in Grenoble.

### Material and Procedure

**FIGURE 1:**
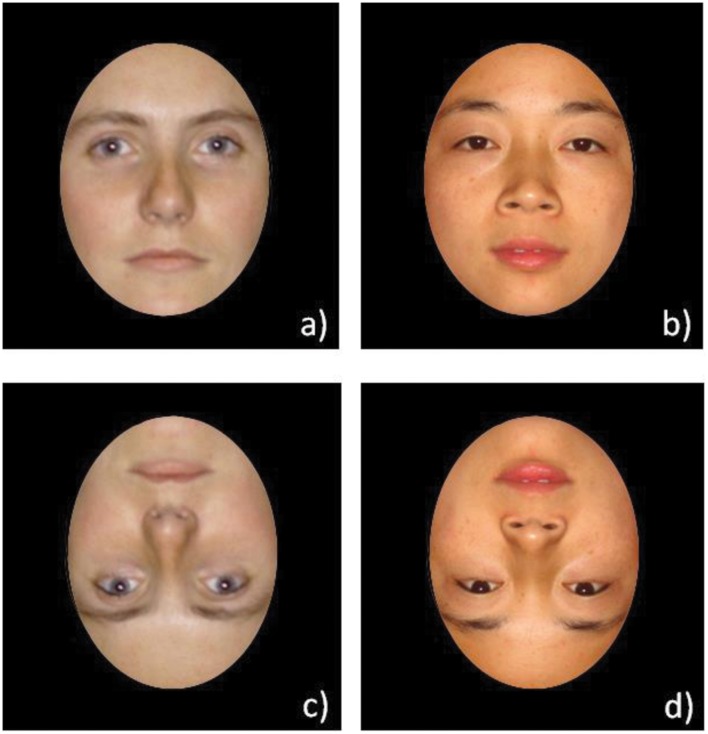
**Example of faces used in the face recognition task: (a) Caucasian face; (b) Chinese face; (c) Inverted Caucasian face (d) Inverted Chinese face**.

There were 20 Caucasian faces (10 of each gender) and 20 Chinese faces (10 of each gender). These faces were selected on the basis of a pre-test conducted with 168 faces where we evaluated typicality, attractiveness, and representativeness of gender. In addition, and as control condition, we used a set of 20 black and white car pictures (10 front view and 10 three-quarter view). The stimuli were standardized and equalized in terms of luminance and contrast. All faces showed a frontal pose and neutral expression (see **Figure 1**). An oval mask was applied on all the faces to limit the use of “external” cues.

We also included a control condition in which the participants had to do exactly the same task as with the faces but with cars (de Heering et al., 2012). They were presented in photographs with a white background. All the pictures (faces and cars) were duplicated and transformed into an inverted version (180° rotation). All the stimuli were displayed on a white background and presented either in upright or inverted orientation. The inversion effect refers to a disproportionate drop in recognition accuracy for inverted faces compared to inverted non-face objects (Yin, 1969). Diamond and Carey (1986) suggested that the configural information required to accurately identify individual faces is disrupted by inversion, forcing a less accurate featural processing strategy (see Maurer et al., 2002 for a definition). Therefore, an inversion effect with facial stimuli is evidence that the face processing system has been engaged. The stimuli were presented in three blocks: Caucasian faces (20 upright and 20 inverted faces), Chinese faces (20 upright and 20 inverted), and Cars (20 upright and 20 inverted).

The experiment was conducted with a 2AFC task. It was programmed with Eprime^®^ software2.0 (Psychology Software Tools, Inc.). The task was displayed by a Monitor LCD Dell (17 inches). A fixation cross appeared for 500 ms and was followed by the face sample on the top of the screen during 500 ms. This face disappeared and 500 ms after, two faces were displayed side by side on the bottom of the screen in the same orientation than the sample (i.e., upright or inverted). One of the faces was the face that was previously presented and another face. The faces remained on the screen until the participant pressed the response key on the computer keyboard. The participants had to press on one key if the face on the left was the same as the first face they saw on the top of the screen or another key if the face on the right was the same as the first face they saw on the top of the screen. The experimenter instructed the participants to base their choice on the global information of the face and not on the faces’ details. They had to answer as fast and accurately as possible. We recorded response time on correct responses (RT) and correct responses (Accuracy). The orientation of the stimuli was distributed randomly in each block. That is, upright and inverted trials were within the same block. The presentation order of the blocks was counterbalanced among participants. The experiment lasted approximately 15 min (instructions and experimentation).

## Results

We used R (R Development Core Team, 2010) for the statistical analyses. We conducted Analyses of Variance (ANOVA) on response time and accuracy with group (Monolinguals, Bilinguals) as between-participants factor and Stimulus type (Caucasian face, Chinese face, Car) and Orientation (Upright, Inverted) as within-participants factor.

### Response Time

**FIGURE 2:**
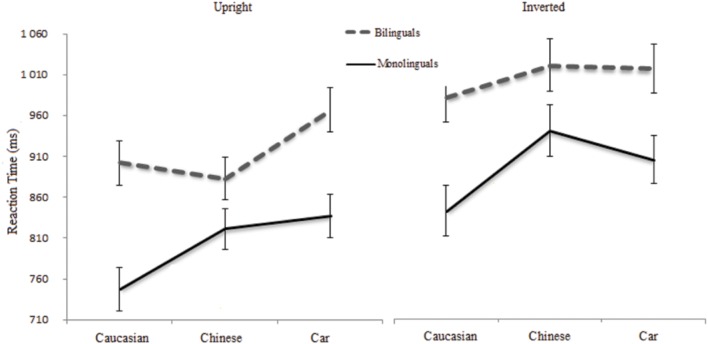
**Mean reaction times (ms) on correct responses for Bilinguals and Monolinguals as a function of stimulus type (Chinese faces, Caucasian faces, cars)**.

**Figure 2** presents the mean response times (ms) on correct responses for Bilinguals and Monolinguals as a function of stimulus type in both orientations. RTs faster than 250 ms and slower than 2500 ms were excluded from the analysis (0.04% of the data). Due to the log-normal distribution of the RT values, we used log transformed [*x* = ln(*x*)] data on the analysis (Csibra et al., 2016). Following the transformation all distributions of RT data were not different from normal according to Shapiro–Wilk normality test.

The analysis revealed that Bilinguals were slower than Monolinguals, *F*_(1,80)_ = 10.53, *p* = 0.002 $\eta_{p}^{2}$ = 0.11. The Stimulus type factor was also significant, *F*_(2,160)_ = 11.81, *p* < 0.001 $\eta_{p}^{2}$ = 0.13. The responses were faster for Caucasian than Chinese faces, *F*_(1,80)_ = 9.91, *p* < 0.01. RTs for the latter and Cars were equivalent *F*_(1,80)_ = 1.68, *p* = 0.19. The Upright orientation yielded faster responses than the Inverted one, *F*_(1,80)_ = 150.17, *p* < 0.001 $\eta_{p}^{2}$ = 0.65. The interaction between Group and Stimulus type was significant, *F*_(2,160)_ = 5.1, *p* = 0.007 $\eta_{p}^{2}$ = 0.06. The interaction between Stimulus type and Orientation was also significant, *F*_(2,160)_ = 11.38, *p* < 0.001 $\eta_{p}^{2}$ = 0.11. The other interactions did not reach significance, *F*s < 1.

Comparisons with a Tuckey-HSD correction showed an Other-Race Effect for Monolinguals in the Upright condition (Caucasian = 747 ms, *SD* = 161 vs. Chinese = 820 ms, *SD* = 161), *p* < 0.001. Furthermore, they discriminated Caucasian faces faster than cars (837 ms *SD* = 181), *p* < 0.001. Their RTs were equivalent for Chinese faces and Cars, *p* = 0.99. For Bilinguals, the RTs for Caucasian (902 ms, *SD* = 175) and Chinese (876 ms, *SD* = 160) faces were equivalent, *p* = 0.82. Car (965 ms, *SD* = 153) processing increased significantly their RTs, compared to Caucasian and Chinese faces, both *p* < 0.001.

In the Inverted condition, Monolinguals also exhibited an Other-Race Effect, since they were faster to discriminate Caucasian faces (843 ms, *SD* = 191) than Chinese faces (941 ms, *SD* = 216), *p* < 0.001). They discriminated Caucasian faces faster than Cars (905 ms, *SD* = 199), *p* < 0.001. Their RTs for Chinese faces and Cars were not significantly different, *p* = 0.57. For Bilinguals, there were no significant differences between the three kinds of stimuli: absence of Other-Race Effect (Caucasian = 979 ms, *SD* = 196 vs. Chinese = 1008 ms, *SD* = 184, *p* = 0.6; Caucasian vs. Cars = 1007 ms, *SD* = 172, *p* = 0.5; Chinese vs. Cars, *p* = 1).

In the Upright condition, Bilinguals’ RTs were slower than Monolinguals for Caucasian faces (*p* = 0.047) but not different for Chinese faces (*p* = 0.9) or Cars (*p* = 0.11). The Bilinguals’ RTs were not affected by the Chinese faces. For the Inverted faces, Bilinguals’ RTs were equivalent than Monolinguals’ for Caucasian faces (*p* = 0.11), Chinese faces (*p* = 0.4), and Cars (*p* = 0.22).

In order to directly test the hypothesis that Monolinguals were more sensitive than Bilinguals to the ORE in the Upright condition we used a planned comparison to test the interaction between Chinese and Caucasian faces and participants (Monolingual/Bilingual). The interaction was significant, *F*(1,80) = 14.27 *p* < 0.001. Monolinguals were slower at processing Chinese faces but Bilinguals processed both face types at the same speed.

### Accuracy

**FIGURE 3:**
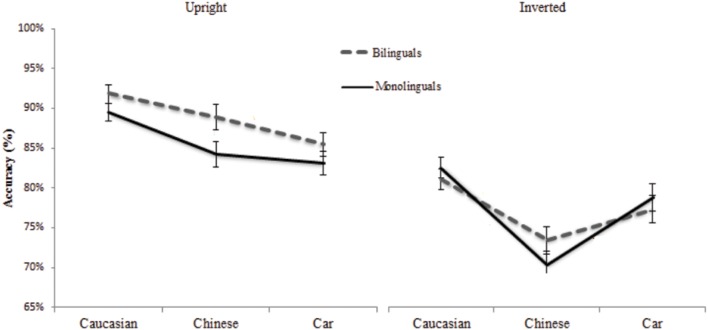
**Percentage of correct responses for Bilinguals and Monolinguals as a function of stimulus type (Chinese faces, Caucasian faces, cars)**.

**Figure 3** presents the percentage of correct responses for Bilinguals and Monolinguals as a function of stimulus type in both orientations. In order to minimize deviation from normal distribution of the accuracy data in percentage, the data were transformed with ArcSin (X^0.5^). The analysis revealed that accuracy was globally equivalent for Bilinguals and Monolinguals, *F*_(1,80)_ = 3.15, *p* = 0.079 $\eta_{p}^{2}$ = 0.03. The Stimulus type factor was significant, *F*_(2,160)_ = 20.07, *p* < 0.001 $\eta_{p}^{2}$ = 0.2. Accuracy was lower for Chinese faces and Cars than Caucasian faces. The orientation effect was large: the Upright orientation yielded higher scores than the Inverted one, *F*_(1,80)_ = 160.23, *p* < 0.001 $\eta_{p}^{2}$ = 0.66. The interaction between Group and Stimulus type was not significant, *F*_(2,160)_ = 1.98, *p* = 0.14 $\eta_{p}^{2}$ = 0.02. The interaction between Group and Orientation was significant, *F*_(2,80)_ = 5.88, *p* = 0.017, $\eta_{p}^{2}$ = 0.066. The interaction between Stimulus type and Orientation was significant, *F*_(2,160)_ = 11.05, *p* < 0.001 $\eta_{p}^{2}$ = 0.12. The other interactions did not reach significance, *F*s < 1.

Comparisons were conducted with the Tuckey-HSD test. The values were corrected for multiple comparisons. The Upright position revealed an Other-Race Effect for Monolinguals, exhibiting higher scores for Caucasian faces (89%, *SD* = 7) than Chinese faces (84%, *SD* = 8), *p* = 0.018. The scores for Caucasian faces were also higher than Cars (83.1%, *SD* = 9), *p* < 0.001. Their accuracy was equivalent for Chinese faces and Cars, *p* = 1. For Bilinguals, the scores for Caucasian faces (91.7%, *SD* = 7) were higher than Chinese faces (88.9%, *SD* = 11), but the difference did not reach significance, *p* = 0.84. The scores for Chinese faces were not significantly higher than Cars (85.4%, *SD* = 9), *p* = 0.2. Accuracy for Caucasian faces was better than Cars, *p* < 0.001.

Planned comparisons showed that accuracy for Caucasian faces was higher than Chinese faces and Cars, *F*_(1,80)_ = 39.16, *p* < 0.001. As in the RT analysis, we compared Monolinguals and Bilinguals on ORE sensitivity. We tested the interaction between Chinese and Caucasian faces, and the two populations. The difference was not significant, *F*_(1,80)_ = 1.16, *p* = 0.28. This null interaction was confirmed in the Upright condition, since accuracy was equivalent for both groups for Caucasian faces, *p* = 0.99. The scores for Chinese faces were higher for Bilinguals than Monolinguals but the difference did not reach significance, *p* = 0.56. For Cars, the differences between the groups were not significant, *p* = 0.99.

In the Inverted condition, Monolinguals exhibited an Other-Race Effect. Their scores were higher for Caucasian than Chinese faces, *p* < 0.001. The scores for Caucasian faces were higher than Cars but the difference did not reach significance, *p* = 0.9. Their scores for Cars were higher than Chinese faces, *p* = 0.003. Bilinguals also exhibited an Other-Race Effect. Their scores were higher for Caucasian than Chinese faces, *p* = 0.023. The scores for Caucasian were higher than Cars, *p* = 0.9.

## Discussion

We hypothesized that early bilingualism could affect the way the cognitive system has specialized –i.e., narrowed– and that this would affect face processing as adults. Previous research revealed that early Bilinguals process audiovisual speech differently than Monolinguals. This study examined whether these processing differences could be due to different ways of processing facial information. To do so, we investigated two well-known effects in face recognition studies: the other-race effect and inversion effect. Bilingual and Monolingual participants did a face recognition task with Caucasian faces (own race), Chinese faces (other race), and Cars that were presented in an Upright or Inverted position. The results revealed that Bilinguals and Monolinguals differed in face recognition processes. For cars, both groups were slower and made more errors compared to faces. The main impact of early bilingual experience seems to be on the temporal domain rather than on accuracy. Bilinguals were globally slower than Monolinguals in their responses. We observed that Monolinguals exhibited the classic Other-Race Effect. Processing Chinese faces (i.e., other race faces) was slower than processing Caucasian faces (i.e., own race faces). For Bilinguals we did not observe an ORE because the processing of Caucasian and Chinese faces were equally time-consuming. Taken together the results indicated that face processing differed between Bilinguals and Monolinguals.

### Differences in Face Processing Speed

The reaction time analysis revealed that Bilinguals were significantly slower than Monolinguals, regardless of the type of stimuli. This pattern of results is in agreement with Haussman et al. (2004) data on cortical organization for face processing. They also observed that Bilinguals were slower than Monolinguals when having to recognize faces that were presented in the left visual field. Data from language studies also revealed processing speed differences between Bilinguals and Monolinguals. Bilinguals were slower than Monolinguals when having to identify audio-visually presented native and non-native phonemes (Burfin et al., 2014). Other results on picture naming tasks reported data that also point to slower processing in Bilinguals than Monolinguals (Costa et al., 2000). The authors accounted for this delay as a supplementary cognitive load –a “bilingual cost”– for lexical processing. Would there be a “bilingual cost” for face processing as well? Bilingualism can indeed affect different kinds of processing that are not necessarily linked to linguistic tasks. For example, bilinguals seem to perform better in tasks that evaluate executive control, attention and solving perceptual conflict situations (Bialystok et al., 2008; Bialystok, 2009; Costa et al., 2009). Further research should be done to address this question of slower reaction times.

Regarding face processing, de Heering et al. (2012) found that deaf adults had slightly higher face recognition scores than the control hearing participants. However, the former were slower to recognize faces. The authors explained their results in terms of early auditory deprivation. However, and as mentioned in the Introduction, these deaf participants were also bilinguals in the sense that they grew up with French lip-reading and sign language. The present results might shed light on a new interpretation. Deaf-bilinguals’ face processing differences might not be linked to deafness only but also to the use of two language codes simultaneously. This hypothesis could be approached by testing native hearing signers. To our knowledge, there is no clear explanation for this “bilingual cost” but there is increasing evidence that bilingual participants are slower in several domains. This issue is of interest and should be investigated in depth.

### Differences on the Other-Race Effect

The present study replicated the Other-Race Effect in Monolinguals for RTs and accuracy. Monolinguals processed faster and better Caucasian than Chinese faces. For Bilinguals instead, the processing of Caucasian and Chinese faces did not yield RT or accuracy differences. In other words, we did not observe an Other-Race Effect on either measure. This result is surprising as the Other-Race Effect is a very classic and robust effect (see Meissner and Brigham, 2001). The differences we observed cannot be accounted for as differences of experience with Chinese faces. The experience with Chinese faces of all the participants –Bilinguals and Monolinguals– was poor and equivalent. Bilinguals were as fast (or as slow) to process both type of faces and their accuracy was equivalent for all the faces. The lack of ORE could have reflected a tradeoff between the speed of their response and accuracy; as they were slower, they might have been a bit more accurate for Chinese faces (decreasing the difference with Caucasian faces). However, as the Bilinguals were even slower for cars and their performance was worse, the tradeoff explanation seems unlikely.

This result may also reflect differences in face processing. Bilinguals might pay attention to other face cues than Monolinguals that could limit the Other-Race Effect. How can contact with a second language affect face processing? Several behavioral studies indicated that the manipulation of face and mouth orientation for example, have an impact on face and visual speech perception. This supports the idea of the involvement of common information processes in face recognition and speech processing mechanisms (Jordan and Bevan, 1997; Rosenblum et al., 2000; for a review see Rosenblum, 2010).

Weikum et al. (2013) and Burfin et al. (2014) reported adult data that comfort the idea that Monolinguals and Bilinguals could use different processing mechanisms to decode visual speech. One might hypothesize that Bilinguals, like de Heering et al.’s (2012) deaf adults, may process faces differently. Indeed, face representation in Bilingual deaf adults and early Bilingual infants do not seem to be biased toward the diagnostic eye region as it is observed in Monolinguals (Lewkowicz and Hansen-Tift, 2012). This could be due to differences in face scanning patterns. This hypothesis should be investigated in future research by examining the temporal scanning patterns of the two populations using static and dynamic faces.

### Inversion Effect

We observed an inversion effect in both groups for accuracy and RT measures, suggesting that all the participants performed configural processing when the faces were presented Upright. If Bilinguals focus on different aspects of the face, it did not affect the classic inversion effect. de Heering et al. (2012) showed an enhanced face inversion effect in the response times of the Bilingual deaf population compared to hearing participants. Based on the perceptual field hypothesis of the face inversion effect (Rossion, 2008, 2009), de Heering et al. (2012) suggested that the hearing participants focus more on the eyes because they are the most diagnostic feature of the face to match inverted faces. Conversely, the Bilingual deaf participants probably needed more time to process the same information. Their representation of a face is generally not as biased toward the diagnostic eye region as it is for hearing participants. The deaf population might focus more on the bottom part of the face than the top because it is the most relevant facial area for decoding phonological cues (e.g., place of articulation that distinguishes /p/ from /t/). It is likely that the Bilinguals in our study behaved the same way as the deaf Bilinguals in de Heering et al.’s (2012) research.

In sum, the main contribution of the present study is that the linguistic experience during the first year of life affects face processing as adults. Bilinguals did not exhibit the classic Other-Race Effect and were slower than Monolinguals during face recognition processes. Early exposure to more than one language leads to a perceptual organization –and therefore narrowing– that seems to go beyond language processing and could extend to the analysis of face configurations. Monolinguals’ face analysis would focus more the diagnostic eye regions. Bilinguals’ face processing strategies would rather be oriented toward the bottom part of the face. Early linguistic exposure could constrain the initial focus of the configural analysis on the lower part of the face, thus delaying the face recognition process. A face recognition eye-tracking study with Bilingual and Monolingual participants would allow us to examine this hypothesis in the future. It is noteworthy that in our study we examined how an individual’s abilities to process other people’s faces can be modulated by his/her linguistic background. We still need to investigate whether an individual’s abilities to process linguistic information can be modulated by his/her experience with face processing. First, infants can be exposed to different kinds of faces (e.g., Caucasian and Chinese) during the perceptual narrowing period. Second, faces convey linguistic cues but also other kinds of non-verbal information that can be very useful for communication.

## Author Contributions

SK: Designed experiment, analyzed data, and wrote the paper. SB: Conducted experiment in Grenoble, contributed in data analysis and paper writing. ER-T: Conducted experiment in Barcelona. DM: conducted the data analysis. AC: Contributed in experiment design and paper writing. OP: Designed experiment, analyzed data, and wrote the paper.

## Conflict of Interest Statement

The authors declare that the research was conducted in the absence of any commercial or financial relationships that could be construed as a potential conflict of interest.

## References

[B1] Benoît C. Mohamadi T. Kandel S. (1994). Effects of phonetic context on audio-visual intelligibility of French. *J. Speech Hear. Res.* 37 1195–1203. 10.1044/jshr.3705.1195 7823561

[B2] Bialystok E. (2009). Bilingualism: the good, the bad, and the indifferent. *Bilingualism* 12 3–11. 10.1139/H07-175

[B3] Bialystok E. Craik F. Luk G. (2008). Cognitive control and lexical access in younger and older bilinguals. *J. Exp. Psychol. Learn. Mem. Cogn.* 34 859–873. 10.1037/0278-7393.34.4.859 18605874

[B4] Burfin S. Pascalis O. Ruiz Tada E. Costa A. Savariaux C. Kandel S. (2014). Bilingualism affects the audio-visual processing of non-native phonemes. *Front. Psychol.* 5:1179. 10.3389/fpsyg.2014.01179 PMC4204456 25374551

[B5] Costa A. Caramazza A. Sebastian-Gallés N. (2000). The cognate facilitation effect: implications for models of lexical access. *J. Exp. Psychol.* 26 1283–1296. 10.1037//0278-7393.26.5.1283 11009258

[B6] Costa A. Hernández M. Costa-Faidella J. Sebastián-Gallés N. (2009). On the bilingual advantage in conflict processing: now you see it, now you don’t. *Cognition* 113 135–149. 10.1016/j.cognition.2009.08.001 19729156

[B7] Csibra G. Hernik M. Mascaro O. Tatone D. Lengyel M. (2016). Statistical treatment of looking-time data. *Dev. Psychol.* 52 521–536. 10.1037/dev0000083 26845505 PMC4817233

[B8] de Heering A. Aljuhanay A. Rossion B. Pascalis O. (2012). Early deafness increases the face inversion effect but does not modulate the composite face effect. *Front. Psychol.* 3:124 10.3389/fpsyg.2012.00124 PMC3336184 22539929

[B9] Diamond R. Carey S. (1986). Why faces are not special: an effect of expertise. *J. Exp. Psychol.* 115 107–117. 10.1037/0096-3445.115.2.107 2940312

[B10] Farah M. J. Wilson K. D. Drain M. Tanaka J. N. (1998). What is “special” about face perception? *Psychol. Rev*. 105 482–498. 10.1037/0033-295X.105.3.482 9697428

[B11] Fort M. Kandel S. Chipot J. Savariaux C. Granjon L. Spinelli E. (2012). Seeing the initial articulatory gestures of a word triggers lexical access. *Lang. Cogn. Process.* 28 1207–1223.

[B12] Fort M. Spinelli E. Savariaux C. Kandel S. (2010). The word superiority effect in audiovisual speech perception. *Speech Commun.* 52 525–532. 10.1016/j.specom.2010.02.005

[B13] Haussman M. Durmusoglu G. Yazgan Y. Güntürkün O. (2004). Evidence for reduced hemispheric asymmetries in non-verbal functions in bilinguals. *J. Neurolinguistics* 17 285–299. 10.1016/S0911-6044(03)00049-6

[B14] Heron-Delaney M. Anzures G. Herbert J. S. Quinn P. C. Slater A. M. Tanaka J. W. (2011). Prevention of the other race effect in infancy via book training. *PLoS ONE* 6:e19858 10.1371/journal.pone.0019858 PMC3097220 21625638

[B15] Jordan T. R. Bevan K. (1997). Seeing and hearing rotated faces: influences of facial orientation on visual and audiovisual speech recognition. *J. Exp. Psychol.* 23 388–403. 10.1037//0096-1523.23.2.388 9104001

[B16] Kelly D. Quinn P. Slater A. Lee K. Ge L. Pascalis O. (2007). The other-race effect develops during infancy: evidence of perceptual narrowing. *Psychol. Sci.* 18 1084–1089. 18031416 10.1111/j.1467-9280.2007.02029.x PMC2566514

[B17] Lewkowicz D. J. Hansen-Tift A. M. (2012). Infants deploy selective attention to the mouth of a talking face when learning speech. *Proc. Natl. Acad. Sci. U.S.A.* 109 1431–1436. 10.1073/pnas.1114783109 22307596 PMC3277111

[B18] Marian V. Blumenfeld H. Kaushanskaya M. (2007). The language experience and proficiency questionnaire (LEAP-Q): assessing language profiles in bilinguals and multilinguals. *J. Speech Lang. Hear Res.* 50 1–28. 10.1044/1092-4388(2007/067) 17675598

[B19] Maurer D. (2015). What atypical adults can teach us about development. *Infancy* 20 587–600. 10.1016/j.pediatrneurol.2014.08.017

[B20] Maurer D. LeGrand R. Mondloch C. J. (2002). The many faces of configural processing. *Trends Cogn. Neurosci.* 6 255–260. 10.1016/S1364-6613(02)01903-4 12039607

[B21] McCullough S. Emmorey K. (1997). Face processing by deaf ASL signers: evidence for expertise in distinguishing local features. *J. Deaf Stud. Deaf Educ.* 2 212–222. 10.1093/oxfordjournals.deafed.a014327 15579849

[B22] Meissner C. Brigham J. (2001). Thirty years of investigating the own-race bias in memory for faces. A meta-analytic review. *Psychol. Public Policy Law* 7 3–35. 10.1037/1076-8971.7.1.3

[B23] Palmer S. B. Fais L. Golinkoff R. M. Werker J. F. (2012). Perceptual narrowing of linguistic sign occurs in the first year of life. *Child Dev.* 83 543–553. 10.1111/j.1467-8624.2011.01715.x 22277043

[B24] Pascalis O. Loevenbruck H. Quinn P. Kandel S. Tanaka J. Lee K. (2014). On the linkage between face processing, language processing, and narrowing during development. *Child Dev. Perspect.* 8 65–70. 10.1111/cdep.12064 25254069 PMC4164271

[B25] Pascalis O. Scott L. S. Kelly D. J. Shannon R. W. Nicholson E. Coleman M. (2005). Plasticity of face processing in infancy. *Proc. Natl. Acad. Sci. U.S.A.* 102 5297–5300. 10.1073/pnas.0406627102 15790676 PMC555965

[B26] Pons F. Bosch L. Lewkowicz D. J. (2015)). Bilingualism modulates infants’ selective attention to the mouth of a talking face. *Psychol. Sci.* 26 490–498. 10.1177/0956797614568320 25767208 PMC4398611

[B27] Pons F. Lewkowicz D. Soto-Faraco S. Sebastián-Gallés N. (2009). Narrowing of intersensory speech perception in infancy. *Proc. Natl. Acad. Sci. U.S.A.* 106 10598–10602. 10.1073/pnas.0904134106 19541648 PMC2705579

[B28] R Development Core Team (2010). *R: A Language and Environment for Statistical Computing.* Vienne: R Foundation for Statistical Computing.

[B29] Rosenblum L. D. (2010). *See What I’m Saying: The Extraordinary Powers of Our Five Senses.* New York, NY: W.W. Norton & Company.

[B30] Rosenblum L. D. Yakel D. A. Green K. P. (2000). Face and mouth inversion effects on visual and audiovisual speech perception. *J. Exp. Psychol.* 26 806–819. 10.1037//0096-1523.26.2.806 10811177

[B31] Rossion B. (2008). Picture-plane inversion leads to qualitative changes of face perception. *Acta Psychol.* 128 274–289. 10.1016/j.actpsy.2008.02.003 18396260

[B32] Rossion B. (2009). Distinguishing the cause and consequence of face inversion: the perceptual field hypothesis. *Acta Psychol.* 132 300–312. 10.1016/j.actpsy.2009.08.002 19747674

[B33] Scott L. S. Monesson A. (2009). The origin of biases in face perception. *Psychol. Sci.* 20 676–680. 10.1111/j.1467-9280.2009.02348.x 19422630

[B34] Sebastián-Gallés N. Albareda-Castellot B. Weikum W. M. Werker J. F. (2012). A bilingual advantage in visual language discrimination in infancy. *Psychol. Sci.* 23 994–999. 10.1177/0956797612436817 22810164

[B35] Sergent J. (1984). An investigation into component and configurational processes underlying face recognition. *Br. J. Psychol.* 75 221–242. 10.1111/j.2044-8295.1984.tb01895.x 6733396

[B36] Sumby W. H. Pollack I. (1954). Visual contribution to speech intelligibility in noise. *J. Acoust. Soc. Am.* 26 212–215. 10.1121/1.1907384

[B37] Weikum W. Vouloumanos A. Navarra J. Soto-Faraco S. Sebastián-Gallés N. Werker J. (2007). Visual language discrimination in infancy. *Science* 316 1159 10.1126/science.1137686 17525331

[B38] Weikum W. M. Vouloumanos A. Navarra J. Soto-Faraco S. Sebastián-Gallés N. Werker J. F. (2013). Age-related sensitive periods influence visual language discrimination in adults. *Front. Syst. Neurosci.* 7:86 10.3389/fnsys.2013.00086 PMC3826085 24312020

[B39] Yin R. K. (1969). Looking at upside-downfaces. *J. Exp. Psychol.* 81 141–145. 10.1037/h0027474

[B40] Zhang S. Morris M. W. Cheng C.-Y. Yap A. J. (2013). Heritage-culture images disupt immigrants’ second-language processing through triggering first-language interference. *Proc. Natl. Acad. Sci. U.S.A.* 110 11272–11277. 10.1073/pnas.1304435110 23776218 PMC3710819

